# Acupuncture Regulates Symptoms of Parkinson’s Disease *via* Brain Neural Activity and Functional Connectivity in Mice

**DOI:** 10.3389/fnagi.2022.885396

**Published:** 2022-06-14

**Authors:** Ju-Young Oh, Ye-Seul Lee, Tae-Yeon Hwang, Seong-Jin Cho, Jae-Hwan Jang, Yeonhee Ryu, Hi-Joon Park

**Affiliations:** ^1^Department of Korean Medical Science, Graduate School of Korean Medicine, Kyung Hee University, Seoul, South Korea; ^2^Studies of Translational Acupuncture Research (STAR), Acupuncture and Meridian Science Research Center (AMSRC), Kyung Hee University, Seoul, South Korea; ^3^Jaseng Spine and Joint Research Institute, Jaseng Medical Foundation, Seoul, South Korea; ^4^Korean Medicine Fundamental Research Division, Korea Institute of Oriental Medicine (KIOM), Daejeon, South Korea

**Keywords:** Parkinson’s disease, acupuncture, functional connectivity, machine learning, primary motor cortex

## Abstract

Parkinson’s disease (PD) is a multilayered progressive brain disease characterized by motor dysfunction and a variety of other symptoms. Although acupuncture has been used to ameliorate various symptoms of neurodegenerative disorders, including PD, the underlying mechanisms are unclear. Here, we investigated the mechanism of acupuncture by revealing the effects of acupuncture treatment on brain neural responses and its functional connectivity in an animal model of PD. We observed that destruction of neuronal network between many brain regions in PD mice were reversed by acupuncture. Using machine learning analysis, we found that the key region associated with the improvement of abnormal behaviors might be related to the neural activity of M1, suggesting that the changes of c-Fos in M1 could predict the improvement of motor function induced by acupuncture treatment. In addition, acupuncture treatment was shown to significantly normalize the brain neural activity not only in M1 but also in other brain regions related to motor behavior (striatum, substantia nigra pars compacta, and globus pallidus) and non-motor symptoms (hippocampus, lateral hypothalamus, and solitary tract) of PD. Taken together, our results demonstrate that acupuncture treatment might improve the PD symptoms by normalizing the brain functional connectivity in PD mice model and provide new insights that enhance our current understanding of acupuncture mechanisms for non-motor symptoms.

## Introduction

Parkinson’s disease (PD) is the second most common neurodegenerative disorder worldwide (Dickson, 2012; Kalia and Lang, 2015; Reich and Savitt, 2019). The pathology of PD is principally characterized by the degeneration of dopaminergic neurons in the substantia nigra (SN; Beitz, 2014), which causes progressive loss of motor symptoms, such as bradykinesia, muscular rigidity, postural instability, or resting tremor (Cacabelos, 2017; Przedborski, 2017). In addition, degeneration of other brain regions causes divers non-motor symptoms, including cognitive deficits, sleep disorders, constipation, and depression, which appear at the premotor stage and often dominate the stages of PD (Barone et al., 2017). Non-motor symptoms have a long-term effect on patients and are closely related to PD pathological processes, thus to profoundly affect the quality of life of patients with PD (Majbour and El-Agnaf, 2017; Schapira et al., 2017). Therefore, as neurodegenerative diseases target large-scale neural networks, it is important to identify the underlying neural mechanisms to manage both motor and non-motor symptoms.

The most common drug for PD, levodopa, has been shown to have severe side effects following long-term administration (Tran et al., 2018). Considering the high prevalance and chronic pathology of PD, an effective treatment regimen with minimal adverse effects is necessary. Acupuncture is one option with a minimal invasion that is currently applied in various forms for patients with PD (Cheng, 2017). Previous studies have shown that acupuncture improves motor function, produces neuroprotective effects in the SN, and suppresses oxidative stress in the striatum (ST; Kim et al., 2011a; Park et al., 2015; Lee et al., 2018). In clinical trials, acupuncture has improved various PD symptoms (Chae et al., 2009; Doo et al., 2015; Fukuda and Egawa, 2015; Cheng, 2017) and patients’ quality of life (Kluger et al., 2016). Furthermore, acupuncture improves not only motor symptoms but also various non-motor symptoms, including cognitive impairment, gastrointestinal dysfunction, depression, and anxiety (Jang et al., 2020; Han et al., 2021). Despite evidence that acupuncture is an effective option for PD, little is known about the regulatory effects of acupuncture on the motor as well as non-motor symptoms and its underlying mechanisms.

The connectome is a graph-theoretical measure that characterizes complex brain neural network topology and quantifies network integration, modularity, or efficient communications using both nodal and global indices. It is an excellent tool for probing the perturbation of structural and functional networks in neurodegenerative disorders (Bullmore and Sporns, 2009; Rubinov and Sporns, 2010), and various types of biomarkers are used including c-Fos. c-Fos is an immediate-early gene used as a surrogate marker for neuronal activity, which enables more direct analyses of neuronal networks. Increased neuronal activity induces upregulation of c-Fos, and subsequent computation of their interregional correlations allows the identification and understanding of a network for the pathophysiology of PD. For instance, SNpc, hippocampus, and hypothalamus in PD patients exhibit both structural and functional alterations in synaptic connections that lead to disconnection, which are correlated to motor and cognition dysfunction (Bellucci et al., 2016). In contrast, the interconnection of regions in the neural system enables high-level performance of the motor and cognition, thus partially explaining mechanisms of the PD treatments, including acupuncture. Moreover, this network may reveal structural and functional differences in the brain regions beyond previous studies in PD.

The 1-methyl-4-phenyl-1,2,3,6-tetrahydropyridine (MPTP) animal model triggers the degeneration of the dopaminergic system, depleting dopamine and its metabolites in the dorsal ST, prefrontal cortex, and hippocampus (Castro-Hernandez et al., 2017). Using this model, we observed the effects of acupuncture on symptoms of PD. Here, we examined whether the brain connectome was impaired in the MPTP model and investigated whether this impairment was improved after acupuncture treatment *via* connectome analysis. In addition, we investigated whether acupuncture treatment controls motor function-related regions as well as other brain regions.

## Materials and Methods

### Animals

Male C57BL/6 mice (weight, 21–25 g; age, 8 weeks; Central Animal Laboratories Inc., Seoul, Korea) were used in this experiment. Mice were raised in constant humidity (60%) and temperature of 23 ± 1°C under a 12/12-h light/dark cycle. Mice were acclimatized with *ad libitum* access to food and water for 1 week prior to the experiment. All procedures were approved by the Kyung Hee University Animal Care Committee for Animal Welfare (KHSASP-19-411) and Dongguk University Animal Care Committee for Animal Welfare (IACUC-2017-020-1), performed according to the guidelines of the National Institutes of Health (NIH) and the Korean Academy of Medical Sciences and followed the recommendations of the International Association for the Study of Pain.

### Drug Treatment

**Figure 1:**
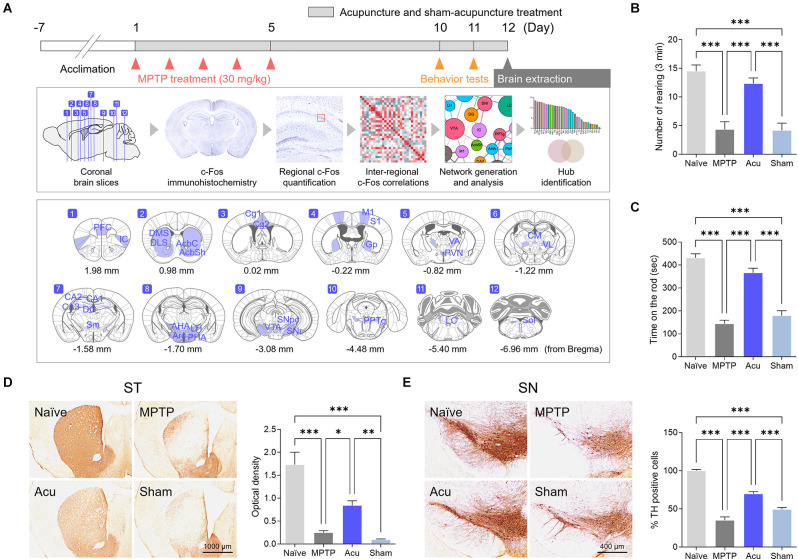
Acupuncture alleviates parkinsonian motor deficits and improves nigrostriatal TH loss in the MPTP-induced PD model. **(A)** Experimental timeline of functional connectivity study. Acupuncture was performed at GB34 for 12 days. Motor behavior tests were performed on days 10 and 11 after the first injection of MPTP (30 mg/kg). To confirm the brain neural responses and brain functional connectivity, the brains were removed 90 min after acupuncture treatment. The c-Fos-positive cells were quantified in each of the 30 brain regions, and functional networks were generated after a series of inter-regional correlations were computed. **(B,C)** Motor behavior evaluated by cylinder and rotarod tests. Acupuncture rescued motor behavior in MPTP-treated animals, but not sham acucpuncture. **(D,E)** Representative images of TH-stained ST and SN tissue and quantification of TH optical density and number of TH-positive cells. Acupuncture markedly alleviates TH loss in ST and TH-positive dopaminergic neurons in the SN of MPTP-treated mice. *n* = 6/group, ***p < 0.001, **p < 0.01, *p < 0.05. All data are mean ± SEM following the one-way ANOVA followed by the Tukey’s test.

Mice were intraperitoneally (i.p.) administered MPTP (30 mg/kg, Sigma-Aldrich, Missouri, USA; 23007-85-4) for five consecutive days to induce PD (Figure 1A). The MPTP treatment procedure was based on that of a previous study (Park et al., 2017).

### Acupuncture Treatment

Acupuncture treatment was performed at bilateral GB34, 2 h after MPTP treatment. Acupoints were selected based on previous studies on PD and were proven to be effective for pain and motor dysfunction (Kim et al., 2005, 2011b; Wang et al., 2011; Yeo et al., 2015). GB34 is located at the hollow point anterior and distal to the fibular head. Sham acupuncture needles (Sham group) were placed on non-acupoints, which were two arbitrary points on the hips for the same period of time as the acupuncture group. Sterilized acupuncture needles (0.18 mm in diameter and 8 mm in length; Haeng-lim-seo-weon Acuneedle Co., Gyeonggi-do, South Korea) were inserted 3 mm deep at each bilateral acupoint and then rotated for 30 s at the speed of two spins per second. One rotation was defined as 180° clockwise and 180° counterclockwise. Mice were rigorously immobilized without anesthesia during acupuncture treatment (Acu group), and treatment was performed accurately and quickly to minimize stress. Naïve mice were immobilized for the same period as acupuncture treatment to induce equal levels of stress.

### Behavior Tests

In the cylinder test, mice were placed in a transparent cylinder (plastic, 12 cm diameter × 20 cm height) and adapted for 1 min before the experiment. After adaptation, the number of rearing sessions was determined. The frequency of contact with the cylinder wall was determined by an observer for 3 min.

For the rotarod test (MED Associates, Inc., St. Albans, VT, USA), mice were pre-trained twice with an accelerating rod for 5 min/day. The running time on the spinning rod was measured; the maximum measurement time was 480 s. The velocity of the rod was gradually increased; the maximum velocity was 40 rpm.

### Immunohistochemistry

Mice were anesthetized and perfused with phosphate-buffered saline (PBS) and paraformaldehyde (PFA) in phosphate buffer (0.2 M). After dehydration, the brains were sectioned at an equal thickness of 40 μm using a freezing microtome (Leica Biosystems, Nussloch, Germany). Sections were cleaned using PBS, activated with 1% hydrogen peroxide (H_2_O_2_) for 15 min, and blocked in a solution containing 0.3% bovine serum albumin (BSA) and 3% Triton X-100 for 1 h. After blocking, the sections were activated using the following primary antibodies: anti-tyrosine hydroxylase (TH; rabbit, 1:1,000 for ST and 1:3,000 for SNpc, Santa Cruz Biotechnology, Santa Cruz, CA, USA; sc-14007) and c-Fos antibody (rabbit, 1:500, Santa Cruz Biotechnology; sc-253). After several rinses, the sections were incubated for 1 h in PBS containing a biotinylated anti-rabbit secondary antibody (Vector Laboratories Inc., Burlingame, CA, USA; BA-1000). Immunoreactions were visible after incubation for 1 h at room temperature in PBS containing avidin-biotinylated peroxidase complex (Vectastain Elite ABC kit; Vector Laboratories Inc.) and in 3-3-diamiobenzidine tetrahydrochloride (Vector Laboratories Inc.; SK-4100). After several rinses, the sections were mounted on slides, dehydrated, and covered with coverslips. Histological images were obtained (Olympus Japan Co., Tokyo, Japan; BX53).

### c-Fos Expressed Cells in Brain Regions

To identify the influence of MPTP and acupuncture on neural activity, c-Fos immunostaining was performed in 30 brain regions. Specific brain regions in which c-Fos-expressing cells were measured are listed in Table 1. Brain regions were defined according to the mouse brain atlas (Paxinos and Franklin, 2008). c-Fos cells were measured thrice in 2.5 × 2.5 mm square in one brain region using Scion Image (Scion Co.), and the mean values were analyzed.

**Table 1:** Abbreviations of 30 brain regions and the number of c-Fos-positive cells in each regions.

			Number of c-Fos positive cells			
No.	Brain region	Abbreviation	Naïve	MPTP	Acu	Sham
	**Cortex**					
**1**	Insular cortex	IC	12.4 ± 0.6	8.7 ± 0.9**	11.0 ± 0.9	9.1 ± 0.2*
**2**	Prefrontal cortex	PFC	17.5 ± 1.1	16.7 ± 0.7	21.7 ± 2.0	20.9 ± 1.4
**3**	Cingulate cortex, area 1	Cg1	11.3 ± 1.7	10.7 ± 1.4	10.0 ± 1.5	9.3 ± 1.3
**4**	Cingulate cortex, area 2	Cg2	12.8 ± 2.3	13.8 ± 1.1	15.6 ± 2.3	12.5 ± 1.0
**5**	Primary motor cortex	M1	31.1 ± 0.5	23.0 ± 1.7**	30.9 ± 1.2^##^	21.1 ± 2^***,$$$^
**6**	Primary somatosensory cortex	S1	36.3 ± 2.2	31.0 ± 2.5	29.0 ± 1.5	28.9 ± 2.4
	**Basal ganglia**					
**7**	Dorsolateral striatum	DLS	5.6 ± 1.0	9.1 ± 0.5	3.9 ± 0.4^*,#^	10.0 ± 1.8^$$^
**8**	Dorsomedial striatum	DMS	8.1 ± 1.2	20.9 ± 4.7*	8.1 ± 1.3^#^	22.0 ± 3.2^*,$^
**9**	Accumbens nucleus core	AcbC	29.5 ± 1.8	38.0 ± 2.9*	26.4 ± 1.2^##^	34.8 ± 1.6^$^
**10**	Accumbens nucleus shell	AcbSh	12.8 ± 1.2	17.9 ± 3.2	8.0 ± 0.6^##^	12.8 ± 1.0
**11**	Globus pallidus	Gp	27.0 ± 2.1	32.9 ± 1.3	20.1 ± 2.5^#^	27.1 ± 4.2
	**Hippocampus**					
**12**	Field CA1 of hippocampus	CA1	19.1 ± 1.6	18.2 ± 1.8	21.8 ± 0.8	19.9 ± 2.1
**13**	Field CA2 of hippocampus	CA2	5.5 ± 0.9	7.5 ± 1.3	6.2 ± 1.1	6.8 ± 0.9
**14**	Field CA3 of hippocampus	CA3	13.4 ± 1.4	12.0 ± 0.6	16.9 ± 0.9^#^	14.2 ± 1.0
**15**	Dentate gyrus	DG	16.7 ± 0.9	13.7 ± 0.6	20.2 ± 1.0^##^	16.1 ± 1.4^$^
	**Thalamus**					
**16**	Ventral anterior thalamic nucleus	VA	6.3 ± 1.4	6.5 ± 0.5	5.5 ± 1.0	5.9 ± 0.5
**17**	Ventral lateral thalamic nucleus	VL	5.6 ± 0.9	5.6 ± 0.6	4.2 ± 0.4	4.3 ± 0.3
**18**	Central medial thalamic nucleus	CM	11.6 ± 1.2	14.1 ± 1.4	6.7 ± 1.5^##^	7.7 ± 1.6^#^
**19**	Submedius thalamic nucleus	Sm	15.9 ± 1.5	20.3 ± 3.3	19.1 ± 2.1	17.9 ± 1.2
	**Hypothalamus**					
**20**	Lateral hypothalamus	LH	23.9 ± 1.2	22.9 ± 0.9	28.7 ± 1.8^*,#^	21.9 ± 0.6^$$^
**21**	Paraventricular hypothalamic nucleus	PVN	57.2 ± 7.5	39.8 ± 7.1	53.8 ± 4.4	42.6 ± 5.4
**22**	Arcuate hypothalamic nucleus	Arc	32.8 ± 6.4	36.8 ± 5.9	26.6 ± 1.0	31.3 ± 2.1
**23**	Anterior hypothalamic area	AHA	30.2 ± 4.0	23.5 ± 4.0	26.2 ± 2.7	26.8 ± 3.2
**24**	Posterior hypothalamic area	PHA	26.9 ± 4.7	24.6 ± 2.4	29.6 ± 2.8	24.2 ± 2.3
	**Midbrain**					
**25**	Ventral tegmental area	VTA	4.1 ± 0.7	5.3 ± 0.6	4.7 ± 0.6	4.0 ± 0.2
**26**	Substantia nigra pars compacta	SNpc	1.3 ± 0.3	2.7 ± 0.2*	1.2 ± 0.2^##^	2.1 ± 0.4
**27**	Substantia nigra reticular	SNr	3.8 ± 0.6	4.8 ± 0.8	4.4 ± 0.5	4.4 ± 1.1
**28**	Pedunculopontine tegmental nucleus	PPTg	10.9 ± 0.8	10.9 ± 0.9	12.5 ± 0.6	12.2 ± 1.4
	**Hindbrain**					
**29**	Locus coeruleus	LC	6.2 ± 0.5	6.6 ± 0.3	7.3 ± 0.6	6.8 ± 0.6
**30**	Nucleus of the solitary tract	Sol	3.7 ± 0.2	4.3 ± 0.5	6.4 ± 0.6^**,#^	5.5 ± 0.5

Brain regions are categorized by major brain subdivisions. *n* = 6/group, ***p < 0.001, **p < 0.01, *p < 0.05 vs. Naïve group, ^##^*p* < 0.01, ^#^*p* < 0.05 vs. MPTP group, ^$$$^*p* < 0.001, ^$$^*p* < 0.01, ^$^*p* < 0.05 vs. Sham group. All data are mean ± SEM following the one-way ANOVA followed by the Tukey’s test.

### Network Generation by Using Gephi

In this study, partial least squares (PLS) analysis was used to evaluate the relationship between c-Fos activity in the brain regions of different groups (Krishnan et al., 2011). Latent variable (LV) pairs were produced for all brain regions investigated in this study, and permutation and bootstrap tests for the LV pairs were conducted to examine whether the effects of the given LV were significantly different from random noise. From the PLS analysis, the correlations between the LVs were abstracted for network analysis with a threshold value of 0.6. Heatmaps were visualized to present the correlation matrix of the LV values between the identified 30 regions.

Within each of the four experimental groups (Naïve, MPTP, Acu, and Sham), networks were generated using the brain connectivity toolbox^1^ and MATLAB. With the brain regions as nodes, the degree and betweenness centralities of all nodes in each network were calculated. Nodes were connected by the edges of interregional correlations produced from the LV pairs in the PLS analysis. The matrices were generated in circular layouts to analyze the overall patterns of functional connectivity between brain regions by group. The matrix was further visualized in a Force atlas layout to analyze the degree and centrality of each brain region represented by nodes. Furthermore, for a more specific investigation of functional connectivity, additional network clusters were generated using only the seven brain regions known to be related to PD from previous studies with super-threshold (>0.5) edges of interregional correlations. The graphical data were visualized using Gephi software (version 0.9.2^2^).

### Machine Learning

Based on the generated network, we developed a machine learning model to predict improvements in motor symptoms using c-Fos and TH levels in the identified neural hubs in the brain identified above. With a limited sample size owing to the experimental nature of the data, the number of features was selected to minimize the sum of errors while reducing the risk of overfitting (Hua et al., 2005). The features were selected to reflect the prediction ability in both the TH levels in regions discussed in previous studies to be related to PD and the c-Fos levels in regions identified by our experiment. The target was the outcomes of the rotarod test conducted in the experiment; therefore, regression models were built.

A total of five algorithms were tested to develop the final model: generalized linear modeling (GLM), extra trees (ET), random forest (RF), gradient boosting method (GBM), and extreme gradient boosting (XGB). The dataset was divided into two sets with a ratio of 0.75:0.25, training and testing set, to learn and validate the model. Five-fold cross-validation was conducted for each model, which divided the data into five subsets and used one subset to test the model trained using the other four subsets. *R*^2^ was used as the metric to measure the goodness-of-fit of each model, and the root mean squared error (RMSE) was used as the metric to avoid overfitting and evaluate the performance of the estimator.

The features and metrics for each model were visualized using a heatmap, and RMSE was treated as a vector and normalized using the L1 norm for the visualization. To express the importance of each feature, the variable importance was treated as a vector and normalized within each model using the L1 norm, where the highest importance was 1. To visually express the relative importance of each variable, the importance above 0.5, was presented in red shades, and those below 0.5 were presented in cyan shades.

The machine learning algorithm was trained and tested using the Scikit-Learn library in Python 3.8.5. The results were visualized using the plot3D package in R version 4.1.2 (2021-11-01)—“Bird Hippie”.

### Statistical Analysis

Statistical analyses were performed using GraphPad Prism 9 (GraphPad Software, Inc., San Diego, CA, USA). Data are presented as mean ± standard error of the mean (SEM). One-way analysis of variance (ANOVA) with Tukey’s test was used to compare groups. Behavioral and c-Fos data were compared using Pearson’s correlation analysis to determine the possible relationships between c-Fos activity and behavioral recovery. The correlation matrix was evaluated using c-Fos expression levels in each of the brain regions and analyzed using a custom Python script. Statistical significance was set at *p* < 0.05.

## Results

### Motor Dysfunction Improvements and Neuroprotective Effects of Acupuncture

To determine whether acupuncture improved motor dysfunction and neuroprotective effects in the MPTP subchronic model as in previous studies, cylinder and rotarod tests were performed, and TH levels were measured in the ST and SN. The cylinder test was measured to evaluate sensory-motor, and the rotarod test was measured to evaluate coordination and balance of motor function.One-way ANOVA showed significant differences among the groups in the cylinder (*F*_(3,16)_ = 22.2, *p* < 0.001) and rotarod test (*F*_(3,16)_ = 36.2, *p* < 0.001). The number of rearing in the MPTP group decreased significantly in the cylinder test (*p* < 0.001 vs. Naïve group, Figure 1B). The time measured on the spinning rod in the MPTP group also decreased significantly in the rotarod test (*p* < 0.001 vs. Naïve group, Figure 1C). In contrast, the Acu group showed recovered functions in the cylinder and the rotarod test (both *p* < 0.001 vs. MPTP group), while the Sham group did not show recovery in the Acu group (*p* < 0.001 vs. Acu group, Figures 1B,C).

In the ST, one-way ANOVA of the optical density values showed significant differences among the experimental groups (*F*_(3,16)_ = 26.6, *p* < 0.001). Similar to previous studies, dopaminergic nerve fibers were significantly decreased in the MPTP group (*p* < 0.001 vs. Naïve group). The Acu group showed recovery in the number of dopaminergic fibers, implying prevention of the destruction of nerve fibers (*p* < 0.01 vs. MPTP group). However, the Sham group did not show any recovery (*p* < 0.01 vs. Acu group, Figure 1D). In the SN, one-way ANOVA of the expression of TH-positive cells also showed significant differences among the groups (*F*_(3,20)_ = 84.6, *p* < 0.001). TH expression was reduced in the PD and Sham groups (*p* < 0.001 vs. Naïve group), whereas acupuncture treatment did not show any reduction (*p* < 0.001 vs. MPTP group, Figure 1E).

### Functional Brain Network After Acupuncture

**Figure 2:**
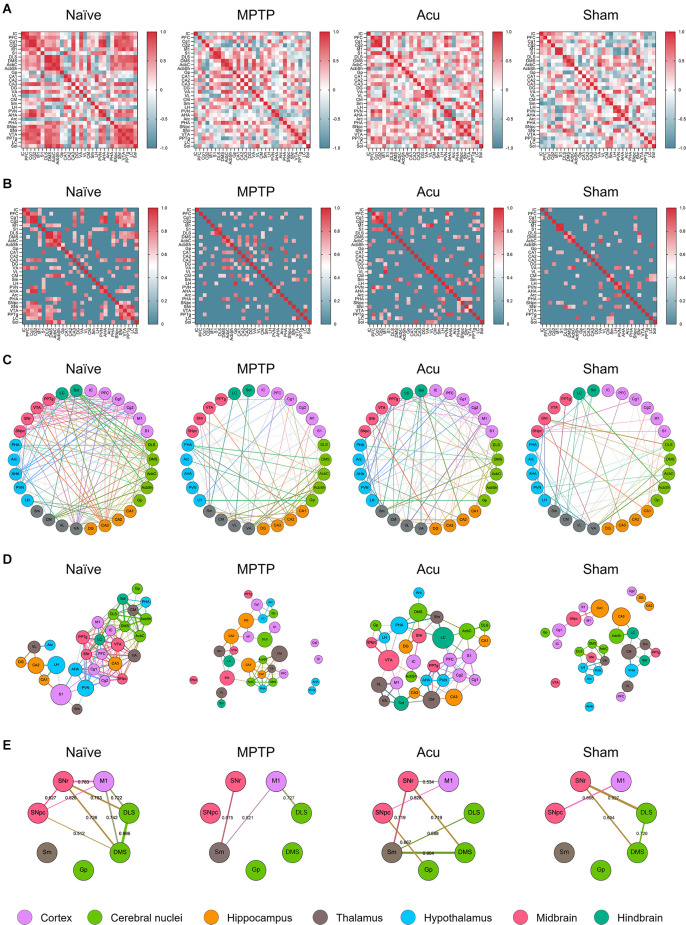
Correlation matrix between regions and clustering of network configurations *via* brain c-Fos expression within each group. **(A)** Color-coded matrices showing inter-regional correlations for c-Fos activation between the 30 brain regions. **(B)** The threshold square of inter-regional correlations for c-Fos activation. MPTP produces a low correlation between the brain regions, and these correlations were altered by acupuncture treatment. The red color indicates high correlation and blue indicates low correlation. **(C)** A circular layout grouped by major brain subdivision to show the connectivity between the brain regions. Nodes are connected by the edges of super-threshold inter-regional correlations. **(D)** A force atlas format to show the degree and betweenness centrality. The number of edges was represented as degree, and the number of shortest paths of all possible pairs of nodes represented betweenness centrality. **(E)** A circular layout grouped by motor-related brain regions.

To investigate functional connectivity between wholebrain regions, as well as the activity of each brain region, inter-regional pairwise correlation values for c-Fos activation among 30 brain regions were computed. The correlation in each group could be expressed *via* the LV matrix (Figure 2A), and the LV matrix was modified to indicate the correlation values over the threshold value of 0.6 (Figure 2B). To analyze the patterns in each group, functional connectivity between the brain regions was generated in a circular layout (Figure 2C). The edges of the clusters showed that the correlation between brain regions decreased overall in the MPTP group compared to that in the Naïve group. Furthermore, subdivided brain regions such as Cg2, IC, AHA, PVN, and SNpc did not make any functional connection with other regions in the MPTP group. However, in the Acu group, the number, and strength of the correlations recovered from the weak correlations in the MPTP group. In addition, the destructed connections of Cg2, IC, AHA, PVN, and SNpc in the MPTP group were restored and reconnected with other brain regions. However, this recovery was not observed in the Sham group. The four putative hub regions of VTA, SNr, LC, and Cg2 were selected as hub regions that reacted to acupuncture treatment (Figure 2C).

### Brain Network Clusters and Functional Connectivity in Force Atlas Format

To identify the degree and centrality of the nodes, the circular layout format was changed to a Force atlas format (Figure 2D). In all results, the nodes were connected by edges, and the weighted number of edges was indicated as a degree. The betweenness centrality for each node is the weighted number of shortest paths that pass through the node from all shortest paths of all possible pairs of nodes. A high degree indicates that one brain region is connected to many other brain regions, and high betweenness centrality indicates that one brain region is functionally correlated with other brain regions. In the Naïve group, the distance between nodes was small, and the correlation between brain regions was high. However, in the MPTP group, some connections between nodes were destroyed, and lower correlation values were measured compared to the Naïve group. In particular, these data showed that subdivided brain regions, such as Cg2, IC, AHA, PVN, and SNpc, did not make any functional connections with other regions. In the Acu group, impaired functional connections between brain regions were recovered. Notably, the connections of the SNpc destroyed by MPTP were restored and interacted with other brain regions.

### Functional Connectivity Between Motor-Related Regions

For a more specific investigation of functional connectivity, seven regions were selected based on previous studies, and correlation values above the threshold of 0.5 were expressed in brain network clusters (Figure 2E). The network clusters showed that in the Naïve group, nine edges were supra-threshold among these brain regions with strong correlations between the midbrain (SNpc and SNr), cortex (M1), and cerebral nuclei (DLS and DMS). The pairs of brain regions with the strongest correlations were the DLS and DMS (*r* = 0.986), SNr and M1 (*r* = 0.783), and M1 and DMS (*r* = 0.742). However, in the MPTP group, only three edges were above the threshold. Correlations between the midbrain, cortex, and cerebral nuclei decreased below the threshold, and the new supra-threshold correlations were observed between the subthalamic thalamus (Sm), midbrain, and cortex. The only remaining correlation in the comparison with the Naïve group was between M1 and DLS (*r* = 0.727). Interestingly, in the Acu group, seven edges were recovered, and correlations between the midbrain (SNpc and SNr), cortex (M1), and cerebral nuclei (Gp and DMS) were observed, in addition to new correlations between the thalamus (Sm) and cerebral nuclei (DLS and DMS). The pairs of brain regions with the strongest correlations were the Sm and DMS (*r* = 0.904), SNr and Sm (*r* = 0.719), and SNr and DMS (*r* = 0.719). This recovery was not observed in theSham group, although four edges survived above the threshold between the midbrain, cortex, and cerebral nuclei.

### Brain Hubs of Node Degree and Betweenness Centrality

**Figure 3:**
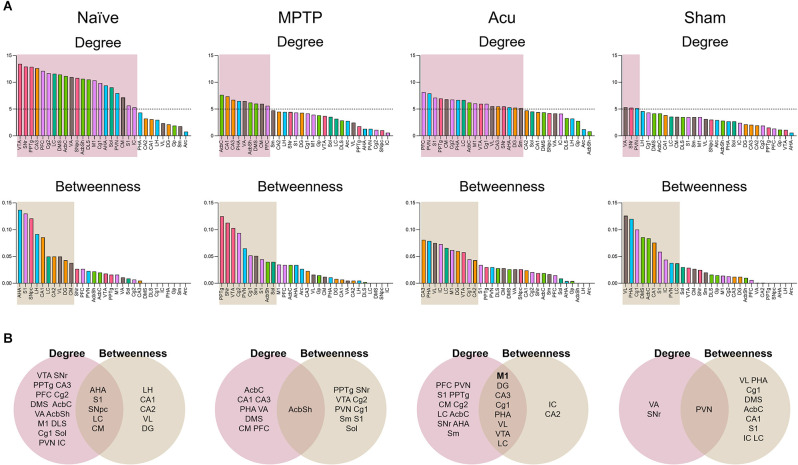
Hub identification of 30 brain regions. **(A)** Brain regions were ranked in descending order for degree and betweenness centrality. Boxes indicated brain regions ranked by a score of 5 or greater for degree and above the 33rd percentile for betweenness. **(B)** The Venn diagram shows the overlap between brain regions ranked for degree and betweenness centrality.

Based on these results, identification of the putative hub regions that work as important brain regions mediating the brain neural mechanism of acupuncture, hub identification was performed (Figure 3A). Brain regions were ranked in descending order of the degree and betweenness of all groups. In the MPTP group, the regions of AcbC, CA1, CA3, PHA, VA, AcbSh, DMS, CM, and PFC were ranked with a score of five or higher for a degree, and PPTg, SNr, VTA, Cg2, PVN, Cg1, Sm, S1, AcbSh, and Sol were ranked-above 10th for betweenness. Subsequently, the overlap of ranked regions was selected. One putative hub region of AcbSh was selected as the hub region that changed after MPTP treatment (Figure 3B). In the Acu group, PFC, PVN, S1, PPTg, CM, Cg2, PHA, LC, AcbC, M1, VTA, Cg1, VL, CA3, SNr, AHA, DG, and Sm were ranked with a score of five or higher, and CA3, PHA, VL, IC, LC, M1, DG, VTA, Cg1, and CA2 were ranked-above rank 10th for betweenness (Figure 3B). These results suggest that in addition to motor function-related areas, other areas also appear as hubs, indicating that acupuncture can control non-motor symptoms in addition to motor function.

### Identifying the Most Significant Brain Region Using Machine Learning Analysis

**Figure 4:**
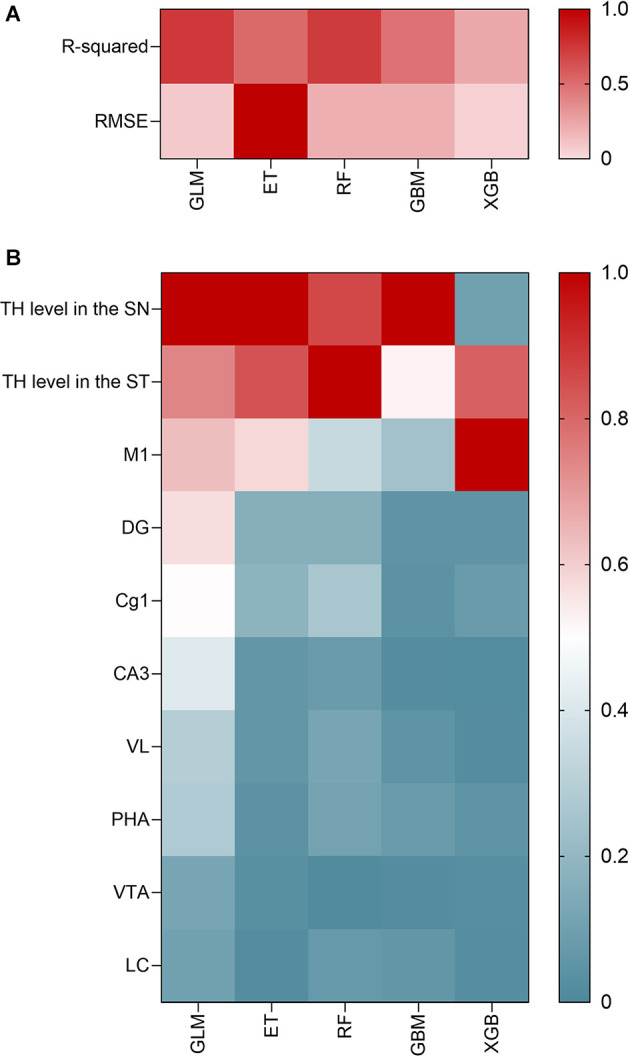
Correlation analysis of machine learning model development and hub areas identified through it. **(A)** Prediction accuracy of the five ML models in terms of the RMSE. **(B)** Important variables through GLM model analysis.

The selected features for developing the machine learning model were c-Fos levels in the identified hub regions: M1, DG, Cg1, CA3, VL, PHA, VTA, and LC, and TH levels in the SN and ST. Among the five models tested for the prediction of rotarod test results in our experiment, the models with the highest *R*^2^ values were GLM (*R*^2^ = 0.727) and RF (*R*^2^ = 0.727), indicating high prediction accuracy. The models with the lowest RMSEs were ET (RMSE = 60.689), GLM (RMSE = 60.99), and RF (RMSE = 62.44). Therefore, the model with both high prediction accuracy and low risk of overfitting in predicting rotarod test results using brain hub regions was GLM followed by RF (Figure 4A).

In the GLM model, the most important variable was TH level in SN (relative variable importance = 1), followed by TH level in ST (relative variable importance = 0.740), and c-Fos level in M1 (relative variable importance = 0.635). The relative proportions of importance were as follows: SNr = 21.3%, ST = 15.7%, and M1 = 13.5%, followed by DG (12.2%), and Cg1 (10.8%). In all five models, the SNr, ST, and M1 were consistently important variables for predicting motor function in PD (Figure 4B). Other hub regions showed a relative variable importance of <0.5.

### Brain Network Centered on M1

**Figure 5:**
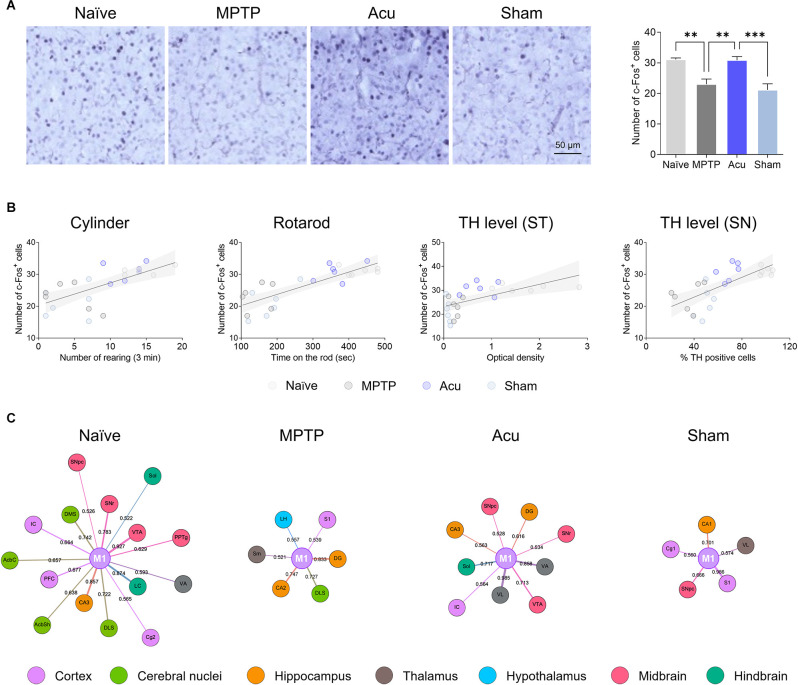
Network generation centered in M1. **(A)** The decreased c-Fos-positive cells in the M1 region of the MPTP-induced PD model were restored by acupuncture treatment. *n* = 6/group, ***p < 0.001, **p < 0.01. Data is mean ± SEM following the one-way ANOVA followed by the Tukey’s test. **(B)** Correlation of c-Fos expression level in the M1 between cylinder, rotarod, TH level in the ST or SN: Cylinder, *r^2^* = 0.5096, *p* < 0.001; Rotarod, *r^2^* = 0.4889, *p* < 0.001; TH level (ST), *r^2^* = 0.3021, *p* = 0.005; TH level (SN), *r^2^* = 0.3918, *p* = 0.001. **(C)** Network analysis centered on M1. Node size is determined by betweenness. Edge betweenness determines the thickness and color intensity of the edges.

Networks with other brain regions were analyzed, with the most significant M1 as the center. One-way ANOVA showed significant differences among the groups (*F*_(3,20)_ = 12.7, *p* < 0.001). c-Fos activity was significantly decreased in the MPTP group (*p* < 0.05 vs. Naïve) and significantly increased by acupuncture stimulation (*p* < 0.05), but not in the Sham group (Figure 5A). Next, the correlation between the behavioral results and TH expression levels in the ST and SN was confirmed (Figure 5B). There was a positive correlation between c-Fos-positive cells and cylinder (*r* = 0.6770, *p* < 0.001), rotarod (*r* = 0.8109, *p* < 0.001), TH level in the ST (*r* = 0.6017, *p* < 0.005), and TH level in the ST (*r* = 0.7055, *p* < 0.001). To identify the connectivity between the M1 and other brain regions, connectivity with other brain regions was confirmed by focusing on the M1 (Figure 5C). As a result of clustering around M1, almost no nodes appeared in the MPTP group compared with the Naïve group. However, since the number of nodes increased after acupuncture treatment, it could be explained that the functional connectivity between M1 and various other regions was restored by acupuncture treatment.

### The Differences in C-Fos Expression Among the Groups

**Figure 6:**
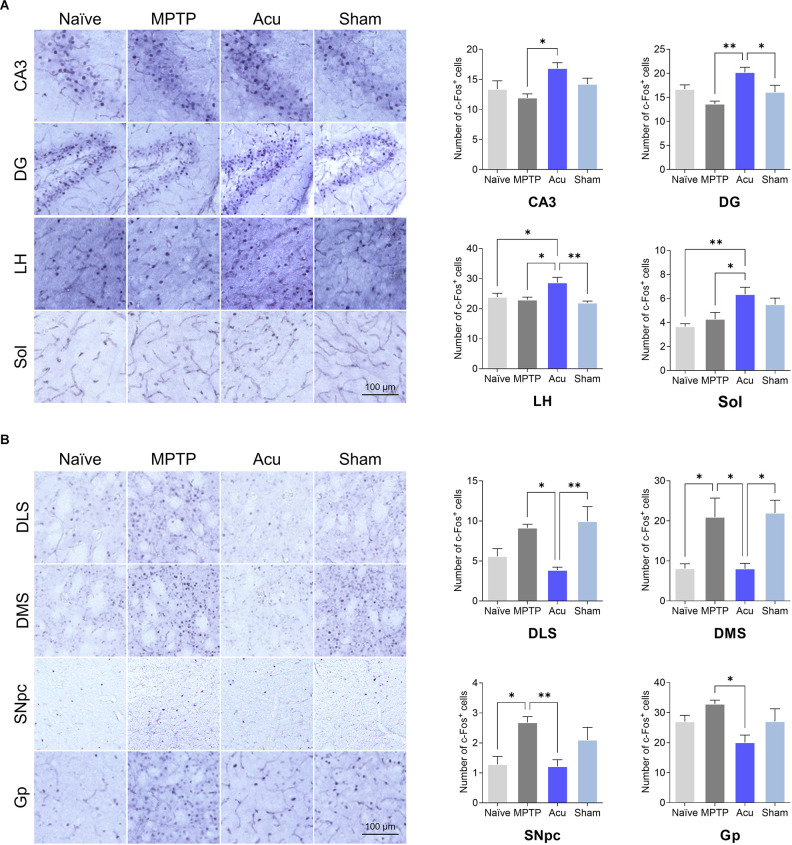
Expressionof c-Fos after acupuncture treatment expressed in each brainregion. **(A)** Brain regions with increased c-Fosactivity after acupuncture. **(B)** Brain regions with decreasedc-Fos activity after acupuncture treatment.*n* = 6/group, **p < 0.01,*p < 0.05. All data are mean ± SEM following the one-way ANOVA followed by the Tukey’s test. CA3, Field CA3 of hippocampus; DG, Dentate gyrus; LH, Lateral hypothalamus; Sol, Nucleus of the solitary tract; DLS, Dorsolateral striatum; DMS, Dorsomedial striatum; SNpc, Substantia nigra parscompacta; Gp, Globus pallidus.

To identify the influence of both MPTP and acupuncture on neural activity in regions other than the M1, c-Fos immunostaining was performed in 30 regions. The number of c-Fos cells increased after acupuncture stimulation in the four brain regions, as shown in Figure 6A. In Acu group, the number of c-Fos-positive cells increased significantly in CA3 (*p* < 0.05 vs. MPTP group), DG (*p* < 0.01 vs. MPTP group), LH (*p* < 0.05 vs. Naïve group, *p* < 0.05 vs. MPTP group), and Sol (*p* < 0.01 vs. Naïve group, *p* < 0.05 vs. MPTP group). The number of DLS and Gp regions increased. In particular, c-Fos activity changes in motor function-related areas. In the MPTP group, the number of c-Fos cells increased significantly in the DMS (*p* < 0.01 vs. Naïve group) and SNpc (*p* < 0.05), and the DLS and Gp regions tended to increase (Figure 6B). In contrast, the number of c-Fos-positive cells in the Acu group decreased significantly in the DLS (*p* < 0.05), DMS (*p* < 0.05), SNpc (*p* < 0.01), and Gp (*p* < 0.05). c-Fos expression in the wholebrain regions is shown in Table 1.

**Figure 7:**
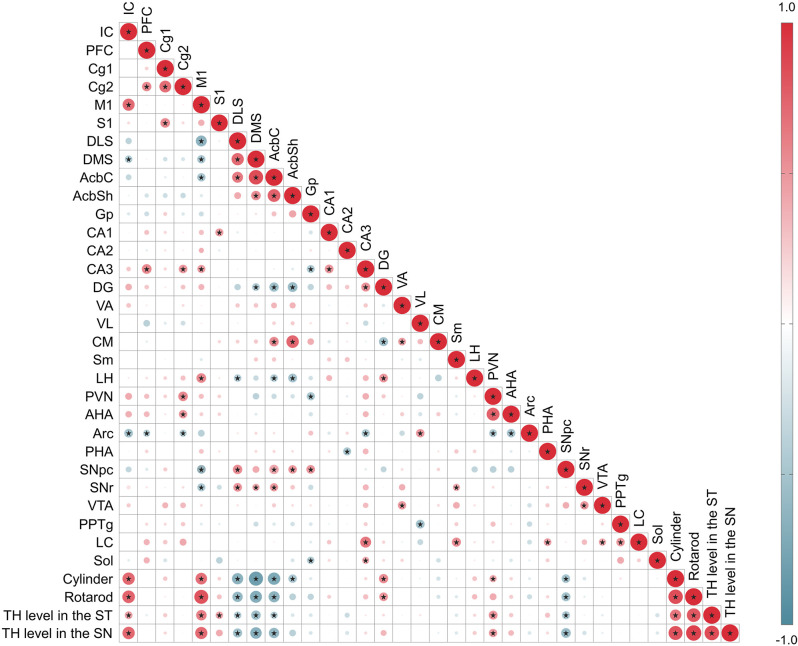
Correlation between c-Fos expression level in 30 regions, motor behavior tests, TH level in the ST or SN. The areas of circles show the value of correlation coefficients. Correlation values are presented in the upper panel in the circle; positive correlations are displayed in red and negative correlations in blue. Color intensity (light to dark) and the size of the circle (small to big) are proportional to the correlation coefficients (0–1 for the positive coefficient and 0 to -1 for negative coefficient) where for example the correlation coefficients on the principal diagonal are equal to 1 (it was represented in dark red and the biggest size of circle). Black stars indicate significant correlations (*p* < 0.001).

Moreover, to understand the functional meaning of the changes in the neural activity of each brain region, Pearson’s correlation analysis was performed using data of motor behaviors, the levels of TH immunoreactivities in the SN and the ST, and the changes of c-Fos in 30 brain regions. Correlation coefficients between parameters were calculated and visualized as heatmaps. Several brain regions were significantly correlated across different other brain regions and motor behaviors. Compared to motor behavior, the IC and M1 regions had a positive correlation, and the DLS, DMS, AcbC, and SNpc regions had a negative correlation. The M1 region was positively correlated with the IC, CA3, and LH regions, and negatively correlated with the DLS, DMS, AcbC, SNpc, and SNr regions, and there was also a marked correlation between motor behaviors and TH levels in the ST or SN (Figure 7).

## Discussion

Here, we investigated the effect of acupuncture on motor abnormalities in PD through brain connectivity in multiple brain regions, using a subchronic animal model of PD. Acupuncture improved motor dysfunction and showed neuroprotective effects on the dopaminergic neurons. Brain connectivity analysis showed that acupuncture treatment induced normalization of impaired brain connectivity in the PD model. In addition, based on machine learning prediction focusing on the hub regions extracted from the connectivity analysis, it was found that M1 is expected to be the most relevant area for PD improvement. Furthermore, the changes of c-Fos in M1 have a positive correlation with TH levels in the SN and the ST and motor-related behaviors. These results suggest that the more the number of c-Fos in the M1, the better improvement of dopaminergic neuronal protection and behaviors.

PD is generally known as a movement disorder caused by the loss of dopaminergic neurons; however, evidence shows that prodromal symptoms begin approximately 10 years before the onset of motor abnormalities and are accompanied by various non-motor symptoms (Poewe et al., 2017). PD affects many brain regions beyond SN and ST, and it has been reported that changes in those brain regions are related to PD pathogenesis which might result in the motor as well as non-motor symptoms. In our recent study, we reported that high levels of α-synuclein and low levels of DJ-1 were observed in the olfactory bulb, hippocampus, amygdala, prefrontal cortex, locus coeruleus, and colon, in addition to the changes of SN and ST in a PD mouse model (Han et al., 2021). These pathological changes in various brain regions were related to non-motor phenotypes such as cognitive impairment, emotional deficit, pain, olfactory dysfunction, and constipation (Han et al., 2021) as shown in patients with PD (Schapira et al., 2017). Furthermore, several studies suggested that brain connectivity among various brain regions changed in PD patients, which might be related to the pathological changes of various brain regions occurring in PD patients. It was reported that functional connectivity was decreased in the thalamic nuclei and associative cortical areas related to the sensorimotor integration or cognitive function in PD patients with freezing of the gate, and the changes in connectivity was associated with disease severity (Wang et al., 2021). In addition, it was also reported that the treatment for PD patients could modify the changed brain connectivity: the subthalamic nucleus-deep brain stimulation showed increased functional connectivity of the supplementary motor cortex, putamen, inferior frontal gyrus, and dorsal frontoparietal network, which was also correlated with changes of UPDRS-III scores (Huang L. C. et al., 2022), indicative of the importance of functional connectivity in the treatment of PD. Neuronal activity-regulated genes, including the prototype immediate early gene, c-Fos, are transiently induced in numerous cell types in many brain regions in response to a wide variety of external stimuli (Greenberg and Ziff, 1984). Thus, c-Fos has been utilized as a key indicator of neuronal responses to various triggers, including specific behavioral and peripheral stimulation, and has been also used for brain-wide maps (Wheeler et al., 2013; Salinas et al., 2018; Chelini et al., 2019; Park et al., 2021; Bonapersona et al., 2022). Here, we also quantified brain neuronal activity by utilizing the c-Fos at the single-cell level. Then, the connectivity of 30 brain regions was computed through graph theory, and potential brain regions associated with acupuncture effects against PD were identified. The brain networks in the MPTP-injected mice were considerably destroyed compared to the Naïve group, and acupuncture treatment induced normalization of the impaired brain connectivity, but not in the sham acupuncture treated group. Network changes were analyzed by focusing on motor-related brain regions. Similarly, although the connections of motor-related brain regions were disrupted in MPTP mice compared to those in the Naïve group, acupuncture treatment improved neural network destruction. This restoration of brain connectivity may be related to the motor function improvement effects of acupuncture. These results suggest the possibility that modulation of brain connectivity can be useful to evaluate the mechanism of therapeutic intervention such as acupuncture in PD.

Next, to further identify the key regions of brain activity, the hub regions in each group were selected through high degree and betweenness centrality using network analysis. A degree is a measure of the number of brain regions connected to a particular brain region to determine the degree of connectivity between them (Higgins et al., 2019). Betweenness is a measure of centrality that indicates the proximity of each brain region connection (Joyce et al., 2010). In this analysis, it was found that the number of hub regions (eight regions) was higher in the acupuncture group than in the MPTP group (two regions). Finally, eight hub brain regions were identified as M1, DG, CA3, Cg1, PHA, VL, VTA, and LC.

Then, to find out the most relevant brain regions among those hub regions, we built machine learning models using the data of c-Fos and TH levels in the brain. This procedure can give us the information as to which brain region could play an important role to predict motor function after acupuncture. Five machine learning models were tested, and the final identified model was the GLM rather than the other four models, which originated from the decision tree models. The results showed that the c-Fos level in M1, along with TH levels in SN and ST, is the most important index among the hub regions related to acupuncture effects, implying the role of M1 in the brain network of PD. There have been several reports showing the importance of M1 in PD (Alexander et al., 1986; Lang and Lozano, 1998; Murer et al., 2002; Dejean et al., 2012; Vitrac et al., 2014). M1, which controls skilled movement, receives inputs from the other cortex and thalamus, and the thalamus integrates data from the basal ganglia and cerebellum to generate output motor commands from layer 5 pyramidal tract neurons, sending convergent signals to connect directly to descending motor pathways (Aeed et al., 2021). Thus, M1 strongly controls movement execution through a central location in the motor circuitry and is critically involved in mediating basal ganglia abnormalities and motor disabilities (Lanciego et al., 2012). Our results showed that the c-Fos expression level in M1 was decreased in the MPTP group compared to the Naïve group, but increased in the acupuncture group, indicating that the decrease in motor function due to the decrease in dopaminergic neurons was improved by acupuncture treatment. This can be interpreted in a similar manner to a previous study where a decrease in the activation of the motor cortex was observed in patients with PD compared to healthy controls (Buhmann et al., 2003; Prodoehl et al., 2010; Spraker et al., 2010; Burciu et al., 2015; Planetta et al., 2015; Mohl et al., 2017). In fact, the c-Fos expression level in M1 and the improvement in motor function analyzed by the rotarod or cylinder test showed a positive correlation, supporting the possibility of predicting improvement in motor function through c-Fos expression in M1. In another study, repetitive transcranial magnetic stimulation of the M1 in patients with PD improves swallowing function (Huang P. L. et al., 2022). Interestingly, in this study, the level of c-Fos expression in M1 was positively correlated with the levels of dopaminergic neurons in the SN and dopaminergic fibers in the ST, as well as motor behavior. On observing a network with other brain regions centered on M1, the number of edges, which was decreased in the MPTP group, increased in the acupuncture group. Moreover, the edges of the acupuncture group showed strong connectivity with the thalamus (VA and VL), hippocampus (DG and CA3), and VTA, which were classified as hub regions. Our previous fMRI study with PD patients also reported that the activity of M1 is related to the improvement of motor function. We performed an fMRI study to observe the neural substrate of acupuncture at GB34 in PD patients. In that study, we calculated the contrast that subtracts the blood-oxygen-level-dependent response for the acupuncture effect by comparing verum acupuncture (VA) and sham acupuncture (SA). The results indicated that the primary motor cortex (M1) was activated when patients with PD received acupuncture treatment (VA vs. SA), which was similar to the present results. Furthermore, the activation of M1 was correlated with individual enhanced motor function (*r* = 0.960; Chae et al., 2009). Taken together, M1 activity through acupuncture stimulation is expected to play an important role in the effects of acupuncture in PD.

We further examine the brain regions in which acupuncture increased the neuronal activity, and found that those of CA3, DG, LH, and Sol were specifically increased by acupuncture. The increasing number of c-Fos-positive cells in the CA3 and DG regions of the hippocampus has been implicated to be related to the increase in hippocampal activity following acupuncture treatment (Squire, 1986). The hippocampus contributes to cognitive function, especially in the construction of the memory system. Hypoactivity of the hippocampus, such as hypometabolism, occurs in PD patients with both motor and cognitive dysfunctions (Gonzalez-Redondo et al., 2014). Previously, c-Fos neural activity was increased in CA3 and DG regions when normal mice were acupunctured, so it can be considered that acupuncture increases c-Fos specifically in these regions regardless of the model (Park et al., 2021). In another study, acupuncture improved affective-related behaviors by modulating protein translations of the mTOR signaling pathway and enhancing pre-and postsynaptic protein levels in the hippocampus in a stress model (Oh et al., 2018). Therefore, the increase of c-Fos activity by acupuncture in these regions might contribute to the improvement of memory and emotional functions in the PD mice model. The literature reveals that the LH is largely populated with orexin- and melanin-concentrating hormone (MCH)-producing neurons, and it was reported that these participated in the neuroprotective action in the dopaminergic neurons (Latifi et al., 2018). In a previous study, orexin and MCH showed neuroprotective effects on dopaminergic neurons, and a massive loss of orexin and MCH neurons was observed in the LH of PD patients (Thannickal et al., 2007). In addition, we found that acupuncture stimulation increased the activation of MCH neurons, which was confirmed by c-Fos and MCH double immunostaining in the LH of PD mice (Park et al., 2017). Furthermore, acupuncture produced the neuroprotective effects in the dopaminergic neurons *via* PI3K/Akt/GSK3β pathways induced by MCH. Thus, it is speculated that the increase of c-Fos positive cells in the LH might be related to the neuroprotective mechanism of acupuncture. Next, the Sol region is the parasympathetic center of the vagal pathway, which transmits a variety of sensory information from the viscera to the brain *via* afferent vagus nerve fibers (Toney and Mifflin, 1994; Boscan and Paton, 2002; Lamy, 2012). It is also connected to the dorsal motor nucleus of the vagus nerve, where the information from various brain regions is integrated to control the viscera through the efferent vagus nerve (Zoccal et al., 2014; Cui et al., 2016; Fang et al., 2017). Recent studies suggested that vagus nerve activation by acupuncture resulted in anti-inflammatory effects through the release of catecholamines from the adrenal medulla (Liu et al., 2021). In other studies, the stimulation of the vagus nerve had neuroprotective effects in the PD model (Farrand et al., 2020). From these perspectives, the elevated levels of c-Fos-positive cells found in the Sol seem to be related to the mechanism of anti-inflammation process against PD pathogenesis (Bonaz et al., 2017; Bai et al., 2019). However, further studies are warranted to reveal the exact mechanism associated with the increase of c-Fos neurons by acupuncture.

And there are some brain regions where the levels of c-Fos were increased in the PD model, whereas those were ameliorated by acupuncture. The number of c-Fos immunoreactivity was increased in the MPTP group in the SN and the ST, which might be related to the pathogenetic process. In contrast, those of the acupuncture group were decreased compared to the MPTP group, indicating the involvement of neuroprotective mechanism by acupuncture. Previously, acupuncture has been reported to have a protective effect on dopaminergic neurons in various animal models of PD, including MPTP, 6-hydroxydopamine, rotenone, and A53T α-synuclein transgenic mice. We also observed that dopaminergic neurons and fibers of the acupuncture group were protected compared to the MPTP group. Regarding this neuroprotective action of acupuncture, several mechanisms have been shown. First, a cell proliferation pathway by neurotrophic factors was presented. Acupuncture has been shown to activate the tropomyosin receptor kinase B-related cell proliferation cascade after increasing brain-derived neurotrophic factors (Park et al., 2003, 2020; Zhao et al., 2019). Glial cell-derived neurotrophic factor and cyclophilin A levels were also significantly increased by acupuncture (Liang et al., 2003; Jeon et al., 2008; Park et al., 2020). Second, acupuncture activated the MCH in the LH involved in neuronal protection by upregulating the downstream pathways related to neuroprotection in the SN (Park et al., 2017). Third, acupuncture also produced antioxidant, anti-inflammatory, and autophagy control (Kang et al., 2007; Tian et al., 2016; Lee et al., 2018). The exact mechanism of decrease of c-Fos in those regions needs to be further elucidated.

This study has several limitations. More brain regions covering wholebrain areas are needed. A method for immunolabeling in wholebrain such as iDISCO can be applicable as a next step. In addition, more specified research is necessary to identify the types of neuronal cells and detailed mechanisms associated with the expressions of c-Fos. Utilizing opto- or chemogenetic studies can be used to elucidate the specific neural mechanism of acupuncture in PD.

In summary, this study showed that acupuncture treatment ameliorated motor dysfunction in PD by restoring the destruction of the brain connectivity in a PD mouse model. network and machine learning analyses revealed that the key region to improve abnormal behavioral functions after acupuncture treatment seems to be related to M1, suggesting that the c-Fos activity of M1 could predict improvement in motor function. Taken together, this study suggests the possibility of improving the effect of acupuncture treatment by normalizing the brain connectivity in PD and suggests the potential role of acupuncture for non-motor symptoms.

## Data Availability Statement

The datasets presented in this study can be found in online repositories. The names of the repository/repositories and accession number(s) can be found in the article.

## Ethics Statement

The animal study was reviewed and approved by Kyung Hee University Animal Care Committee for Animal Welfare and Dongguk University Animal Care Committee for Animal Welfare.

## Author Contributions

H-JP, J-YO, and T-YH conceived and designed the study. J-YO, T-YH, and J-HJ performed experiments. J-YO, Y-SL, T-YH, and S-JC analyzed and interpreted the data. J-YO, Y-SL, T-YH, and S-JC developed the methodology. H-JP and YR administrated the technical or material support. J-YO and Y-SL wrote the draft. H-JP supervised the study. All authors contributed to the article and approved the submitted version.

## Conflict of Interest

The authors declare that the research was conducted in the absence of any commercial or financial relationships that could be construed as a potential conflict of interest.

## Publisher’s Note

All claims expressed in this article are solely those of the authors and do not necessarily represent those of their affiliated organizations, or those of the publisher, the editors and the reviewers. Any product that may be evaluated in this article, or claim that may be made by its manufacturer, is not guaranteed or endorsed by the publisher.

## Footnotes

^1^ http://www.brain-connectivity-toolbox.net/

^2^ https://gephi.org/
